# Purple Urine Bag Syndrome in a Young Adult With Neurogenic Bladder Secondary to Partial Caudal Regression Syndrome: A Case Report

**DOI:** 10.7759/cureus.98197

**Published:** 2025-11-30

**Authors:** Suryakanta Seth, Abhimanyu Vasudeva, Dharmendra K Pipal, Ravi Gupta, Gaurav Gupta

**Affiliations:** 1 Anatomy, All India Institute of Medical Sciences, Gorakhpur, Gorakhpur, IND; 2 Physical Medicine and Rehabilitation, All India Institute of Medical Sciences, Gorakhpur, Gorakhpur, IND; 3 General Surgery, All India Institute of Medical Sciences, Gorakhpur, Gorakhpur, IND; 4 Surgery, All India Institute of Medical Sciences, Gorakhpur, Gorakhpur, IND

**Keywords:** case report, caudal regression syndrome, long-term catheterization, neurogenic bladder, purple urine bag syndrome

## Abstract

Purple urine bag syndrome (PUBS) is a rare, benign condition associated with long-term urinary catheterization, caused by bacterial metabolism of urinary indoles into blue and red pigments. We present the case of a 22-year-old female with a neurogenic bladder secondary to partial caudal regression syndrome who developed PUBS. The patient presented with a history of purple discoloration of her catheter bag. She had been on continuous bladder drainage since age three, with routine catheter changes every two weeks. Management involved patient reassurance and emphasis on catheter hygiene. A permanent surgical solution for her underlying neurogenic bladder was not pursued after a multidisciplinary review concluded that available options were either anatomically nonviable or unacceptable to the patient. This case highlights the importance of recognizing PUBS to alleviate patient anxiety, provides a clear rationale for conservative management, and underscores the challenges of managing chronic bladder dysfunction in patients with complex congenital anomalies. The patient provided broad written informed consent for publication.

## Introduction

Purple urine bag syndrome (PUBS) is a rare and visually striking condition characterized by purple discoloration of urine in catheter drainage systems. It is typically associated with long-term catheterization, female sex, and urinary tract colonization by bacteria such as *Providencia stuartii*, *Klebsiella pneumoniae*, and *Escherichia coli* [1,2]. Recognition is important because PUBS is generally benign, though it can cause significant concern for patients and caregivers [1]. While most reported cases occur in elderly, institutionalized patients, PUBS is an important diagnostic consideration in any chronically catheterized individual, including younger patients with congenital neurogenic bladder.

Caudal regression syndrome (CRS) is an uncommon congenital disorder characterized by partial or complete absence or malformation of the caudal vertebrae, frequently accompanied by spinal cord anomalies, with an estimated incidence of 0.1 to 0.25 per 10,000 live births [3,4].

The urinary bladder originates from the endodermal urogenital sinus around the fourth week of gestation, following the division of the cloaca by the urogenital septum. The upper portion of the sinus forms the bladder, while its distal part develops into the urethra. Incorporation of the mesonephric ducts contributes to the formation of the trigone, and the ureteric buds eventually acquire separate openings into the bladder [5]. Functionally, bladder activity depends on coordinated input from parasympathetic (S2-S4), sympathetic (T10-L2), and somatic (S2-S4) nerves that regulate detrusor contraction, outlet resistance, and voluntary sphincter control [6]. Disruption of these developmental or neural pathways, as seen in CRS, results in a neurogenic bladder characterized by impaired storage and voiding functions due to altered neural control.

CRS represents a heterogeneous condition in both its etiology and developmental mechanisms, likely arising from a combination of genetic susceptibility and environmental influences [7]. Moreover, genetic studies have identified autosomal dominant sacral agenesis linked to the 7q36 locus and mutations in the homeobox gene HLXB9, underscoring a molecular basis for some instances of CRS [8,9].

This case report presents a 22-year-old female with long-term catheterization due to partial sacral agenesis (CRS) who developed PUBS. The report aims to illustrate the clinical approach to PUBS, discuss the long-term management challenges in adults with congenital neurogenic bladder, and emphasize the ethical importance of aligning care with patient-defined goals.

## Case presentation

A 22-year-old female with a neurogenic bladder secondary to partial sacral agenesis (CRS) presented after noticing a purple discoloration of her Foley catheter bag two days prior. A neurogenic bladder, a functional disturbance of urinary storage and emptying resulting from impaired neural control, often necessitates long-term catheterization and predisposes to recurrent infections. She had been on continuous bladder drainage since early childhood, with routine catheter changes every two weeks under aseptic precautions. She reported no motor weakness, no sensory deficits (limited assessment due to patient preference), and no bowel involvement. Previous records indicated hydroureteronephrosis since childhood, consistent with longstanding bladder dysfunction. A maternal history of diabetes mellitus was not documented.

The patient had previously been under follow-up at other hospitals before presenting to our institution, a tertiary hospital in Northern India. She was well-informed about her condition and clear about her expectations regarding management. Her presenting concerns were twofold: the acute issue of purple discoloration of her catheter bag and her long-term goal of achieving a permanent solution for her bladder dysfunction.

**Figure 1:**
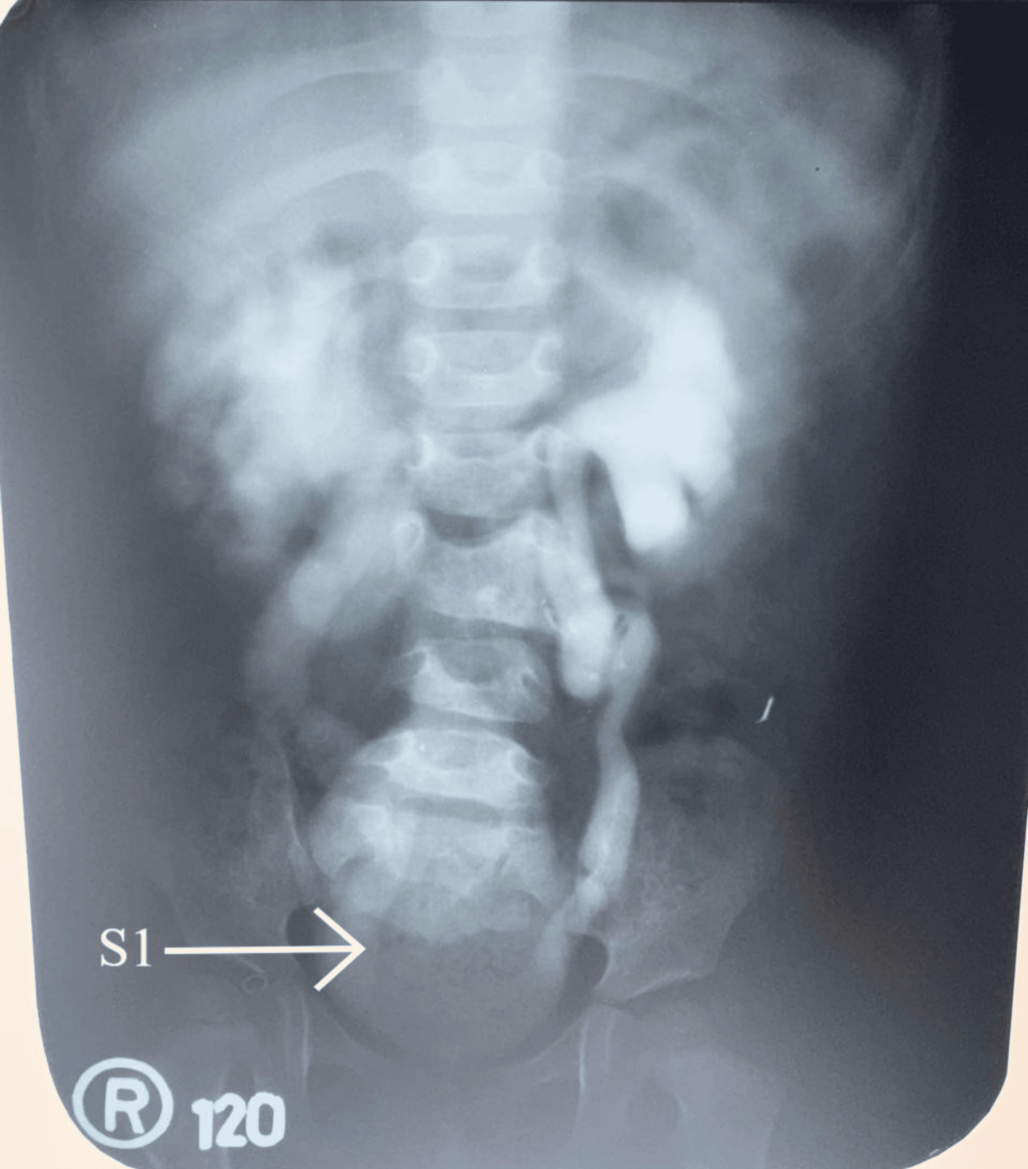
Pelvic X-ray (PA view) showing distal sacral agenesis Pelvic X-ray (anteroposterior view, bladder filling phase) obtained in early childhood demonstrating non-visualization of the distal sacral and coccygeal vertebrae. The arrow indicates the abrupt truncation of the sacrum, while the S1 vertebral segment is clearly delineated for reference. Film coverage was adequate, and there was no obscuration by bowel gas. These findings are consistent with distal sacral agenesis. PA: posteroanterior

During early childhood, prior to initiation of long-term bladder drainage, a pelvic X-ray was performed with parental consent. The X-ray (bladder filling phase of a micturating cystourethrogram, PA view) demonstrated non-visualization of the distal sacral and coccygeal vertebrae, with only the S1 vertebral segment clearly delineated (Figure 1).

Film coverage was adequate, and there was no obscuration by bowel gas. The abrupt truncation of the sacrum was thus considered genuine rather than technical, supporting the diagnosis of distal sacral agenesis. These findings are radiologically compatible with CRS [10].

Archival radiographs and CT scans from childhood were reviewed. These images were digitized from original X-ray films by photographing them on a view box, as the original digital data are unavailable. Imaging was performed nearly two decades ago on conventional film-based and early-generation CT systems, when digital archiving was, to the best of our knowledge, difficult to trace. Comprehensive documentation of her treatment continuum was available, and the prior imaging adequately demonstrated relevant anatomical findings. Repeat imaging was not clinically indicated, in accordance with the ALARA principle, and the patient provided informed consent for inclusion of anonymized archival images. Given her stable clinical status, absence of new urinary or systemic symptoms, and preference to avoid further testing, repeat studies were not pursued. The available archival radiographs adequately demonstrated the underlying anatomical abnormality, and additional imaging was not expected to alter management.

In the current presentation, the patient was afebrile, hemodynamically stable, and well-nourished. The catheter and urine bag showed striking purple discoloration, with turbid urine. No suprapubic or flank tenderness was noted. The patient declined a full neurological examination due to privacy concerns. A photograph of the discolored urine bag was not obtained, as the patient declined photography; however, the treating team directly observed and documented the characteristic purple hue at the time of presentation. She was independently ambulant and reported regular bowel movements without constipation.

Although urinalysis and culture were ordered, the patient was managed conservatively with reassurance and catheter hygiene during her initial visit. As she remained afebrile and asymptomatic, this approach aligned with guidelines for asymptomatic bacteriuria, making the pursuit of test results unnecessary for clinical decision-making.

The patient was counseled regarding the etiology of the purple discoloration of the urine bag and appropriate catheter care. Her long-term management goals, including options for achieving a permanent solution for her bladder dysfunction, were discussed in detail. Clean intermittent catheterization (CIC) was discussed with the patient as a long-term management option. However, she declined CIC because she had been on continuous indwelling catheter drainage since early childhood and found the idea of frequent self-catheterization unacceptable. Given her prior follow-ups at other institutions, a coordinated care plan was established to ensure continuity while avoiding unnecessary investigations. Since the patient was clinically stable, afebrile, and without any new urinary or systemic symptoms suggestive of upper tract deterioration. In view of her longstanding continuous bladder drainage and preference to avoid further testing, repeat ultrasonography of the kidneys, ureters, and bladder, or urodynamic studies, was deferred, as these were not expected to change clinical management.

Written informed consent was obtained from the patient for publication of this case report and any accompanying images, including the radiographic image acquired during her childhood. The consent form explicitly covered discussion of her clinical management, imaging findings, and her expressed perspective on her care.

## Discussion

This case offers a valuable clinical lesson in managing a dramatic but benign condition (PUBS) within the complex context of a lifelong congenital disorder (CRS). The pathophysiology of PUBS is well established, involving bacterial metabolism of dietary tryptophan into indole, its hepatic conversion to indoxyl sulfate, and subsequent breakdown by urinary tract sulfatases into the pigments indigo (blue) and indirubin (red) [11]. While most reported cases occur in the elderly, our case demonstrates that the syndrome can manifest in any patient with long-term indwelling catheter use [12].

The mainstay of PUBS management comprises reassurance, catheter replacement, and maintenance of proper hygiene. Empiric antibiotics are generally unnecessary unless there is clinical evidence of infection [13]. Our patient was afebrile, hemodynamically stable, and had no localizing symptoms. Initiating antibiotics would have contravened guidelines for asymptomatic bacteriuria, which strongly recommend against treatment in catheterized patients to avoid promoting antimicrobial resistance without clinical benefit [14].

A central challenge in this case was addressing the patient’s desire for a “permanent solution.” A thorough review of surgical options was conducted. Bladder augmentation or the creation of a continent catheterizable channel (Mitrofanoff) would still necessitate lifelong CIC, which the patient found unacceptable. The most definitive procedure, an ileal conduit urinary diversion, would eliminate catheters but requires a permanent abdominal stoma; the patient declined this due to its irreversibility and impact on body image. Furthermore, sacral nerve stimulation was not viable due to the congenital absence of the sacral vertebrae, which implies the concomitant absence of the requisite sacral nerve roots (as demonstrated on the early childhood X-ray, Figure 1). Therefore, management consensus focused on optimizing her current care, aligning with her preference to avoid major irreversible surgery. The observed discoloration was consistent with PUBS, a benign, self-limiting phenomenon associated with long-term catheterization. Although follow-up was not available to document color resolution, the clinical presentation and exclusion of alternative causes supported this diagnosis. Additionally, previous multidisciplinary reviews had concluded that surgical or continent diversion options were either anatomically non-viable or inconsistent with her preferences. Therefore, conservative management with continued aseptic catheterization, hygiene reinforcement, and counseling was pursued in alignment with her informed choice and quality-of-life priorities.

This case highlights several key lessons. First, PUBS is a benign sentinel sign, not an emergency. Second, managing adults with congenital conditions requires a nuanced understanding of surgical options and a deep respect for patient preferences and quality of life. Finally, it underscores the ethical imperative of broad, informed consent when sharing clinical lessons that include patient perspectives.

This case may also serve as a reference point for clinicians encountering similar presentations, aiding in timely recognition, patient reassurance, and appropriate management.

## Conclusions

This case of PUBS in a patient with CRS serves as a reminder that benign, treatable conditions can coexist with complex, chronic disabilities. The management of such patients must be holistic, prioritizing patient reassurance, hygiene, and quality of life over aggressive, unnecessary treatment. Clinical decisions must be rooted in a clear risk-benefit analysis and adherence to evidence-based guidelines. The patient remained clinically stable at presentation, with no evidence of acute deterioration. However, follow-up documentation was not available to assess subsequent clinical progression. Future efforts should focus on improving structured transition care for young adults with congenital anomalies, ensuring they are supported through lifelong management decisions. This case also demonstrates the critical role of transparent communication and broad, informed consent in ethically sharing clinical lessons to advance medical knowledge.
